# Is Clinical Remission, an Ambitious Treatment Goal, Achievable in Patients with Moderate-to-Severe Asthma on Inhaled Therapies: How Ambitious Should We Be?

**DOI:** 10.3390/jcm15041497

**Published:** 2026-02-14

**Authors:** Soichiro Hozawa, Risako Ito, Jodie Crawford, Ryota Hibi, Alison Moore, Stephen G. Noorduyn

**Affiliations:** 1 Hiroshima Allergy and Respiratory Clinic, Hiroshima 730-0013, Japan; 2 Respiratory/Immunology, Japan Real-World Evidence & Health Outcomes Research, GSK, Tokyo 107-0052, Japan; 3 Development Biostatistics, GSK, London WC1A 1DG, UK; 4 Respiratory Medical Affairs, GSK, Tokyo 107-0052, Japan; 5 Global Medical Affairs, General Medicines, GSK, London WC1A 1DG, UK; 6 Global Real-World Evidence & Health Outcomes Research Organization, GSK, Mississauga, ON L5R 4H1, Canada; 7 Department of Health Research Methods, Evidence and Impact, McMaster University, Hamilton, ON L8S 4L8, Canada

**Keywords:** asthma, clinical remission, inhaled therapies, Japan, Asthma Prevention and Management Guideline (JGL), moderate asthma, post hoc analysis, Practical Guidelines for Asthma Management (PGAM), severe asthma, single-inhaler triple therapy

## Abstract

**Background/Objectives**: Clinical remission (CR) is an ambitious and attainable treatment goal for asthma; however, CR definitions vary. Evidence of CR in Japanese patients with moderate-to-severe asthma on inhaled therapies is lacking and was evaluated based on three guideline definitions: the United States Workgroup consensus statement, Japanese Guidelines for adult asthma (JGL), and Practical Guidelines for Asthma Management (PGAM). **Methods**: Post hoc analysis of Phase III studies including Japanese participants: Japanese subpopulation of CAPTAIN (NCT02924688) and a 52-week Japanese long-term safety study (NCT03184987). CAPTAIN randomized participants to once-daily fluticasone furoate/vilanterol (FF/VI) regimens ± umeclidinium (UMEC). The long-term safety study allocated participants to once-daily FF/UMEC/VI based on asthma control status. All three CR definitions assessed systemic corticosteroid use, severe exacerbations, and asthma control (Asthma Control Questionnaire-5 <1.5 [Workgroup] or ≤0.75 [JGL/PGAM]); Workgroup and JGL also assessed lung function (change from baseline in trough forced expiratory volume in 1 s of ≥0 [stabilized] or ≥100 mL [optimized]). **Results**: CR attainability varied on definition and thresholds used. At Week 24 in the CAPTAIN Japanese subpopulation, 34–59% and 18–45% of participants (Workgroup; stabilized and optimized), and 21–34% and 8–24% (JGL; stabilized and optimized) met CR criteria across treatment arms. At Week 52 in the long-term safety study, equivalent figures for CR achievement were 33–60%, 22–45%, 11–28%, and 11–23%. **Conclusions**: This analysis demonstrates that CR, using different definitions and criteria, is an attainable treatment goal with inhaled therapy in Japanese patients with moderate-to-severe asthma not yet eligible for biologics.

## 1. Introduction

Asthma affects approximately 5 million people in Japan, and it is a leading chronic respiratory disease both globally and nationally [1]. A nationwide Japanese descriptive study reported that approximately 45% of patients with severe asthma aged ≥12 years included in the National Database of Health Insurance Claims and Specific Health Checkups were considered to have uncontrolled symptoms, based on European Respiratory Society/American Thoracic Society guidelines criteria [2,3].

Currently recommended pharmacological treatment options for asthma in Japan are generally aligned with those in the Global Initiative for Asthma guidelines [4]. Inhaled corticosteroid/long-acting β_2_-agonist (ICS/LABA) combination treatment is a cornerstone of the long-term treatment of asthma. The Japanese Guidelines for adult asthma (JGL) recommend mid- or high-dose ICS, with the addition of a LABA and/or a long-acting muscarinic antagonist (LAMA) in a stepwise approach for asthma symptoms that remain uncontrolled with ICS alone [5,6]. Practical Guidelines for Asthma Management (PGAM), developed by the Japan Asthma Society, recommends initiating treatment with mid-dose ICS/LABA for patients with mild-to-severe asthma [7].

Clinical remission (CR) is increasingly being recognized as an ambitious and attainable treatment goal for patients with asthma [8,9], which is supported by treatment guidelines [6,7]. Currently, there is no standardized definition of CR in asthma. While definitions vary between guidelines, four criteria are typically used to assess CR, including absence of symptoms (e.g., Asthma Control Test [ACT] score ≥20 or Asthma Control Questionnaire [ACQ] <1.5), no exacerbations, no use of systemic corticosteroids (SCS), and optimized/stabilized lung function, while complete remission includes additional normalization of underlying pathology [9,10]. In a United States (US) Workgroup (including members from the American College of Allergy, Asthma, and Immunology, the American Academy of Allergy, Asthma, and Immunology, and the American Thoracic Society) consensus statement, in addition to the four key criteria, additional criteria include no missed work/school, continued use of controller therapies at low–medium ICS dose, and rescue therapy use no more than once per month [8,9,11].

In Japan, JGL 2024 defines CR using four components: regular oral corticosteroid (OCS) use, exacerbation frequency, symptom control, and lung function [6], whereas PGAM 2024 excludes lung function from its definition [7].

Single-inhaler triple therapy with fluticasone furoate/umeclidinium/vilanterol (FF/UMEC/VI) is approved in Japan as a once-daily maintenance treatment for adult patients with uncontrolled asthma, based on positive findings from the pivotal randomized Phase IIIa CAPTAIN study (NCT02924688) that demonstrated the efficacy and safety of FF/UMEC/VI versus FF/VI in patients with asthma inadequately controlled on ICS/LABA [12,13]. Similar results were also observed in the subgroup of Japanese participants enrolled in the CAPTAIN study [13]. Moreover, a non-randomized Phase III study (NCT03184987) evaluating the long-term (52 weeks) safety of fixed-dose FF/UMEC/VI in Japanese patients with asthma found no additional safety concerns in this population [14].

While CR has been studied in patients with severe asthma eligible for biologics [9,15], and data on CR in patients with moderate asthma treated with inhaled therapies not yet eligible for biologics are emerging [16], there is little evidence for CR in Japanese patients with asthma. This post hoc analysis evaluated CR in Japanese patients with moderate-to-severe asthma referencing either the US Workgroup consensus statement, JGL, or PGAM definitions. Data were drawn from two Phase III studies incorporating Japanese participants: the Japanese subpopulation of the CAPTAIN trial [12] and the 52-week Japanese long-term safety study [14].

## 2. Methods

### 2.1. Ethics

The CAPTAIN Japanese subpopulation and long-term safety studies [12,13,14] were conducted in accordance with the Declaration of Helsinki, International Conference on Harmonisation Good Clinical Practice guidelines, and applicable country-specific regulatory requirements. The protocol was reviewed and approved by an internal GSK review board and received approval from central or local institutional review boards (IRBs) or independent ethics committees. All participants provided written informed consent, and participant anonymity was preserved using methods approved by the IRB.

### 2.2. Study Design

This post hoc analysis evaluated CR in patients with moderate-to-severe asthma using data from two studies [13,14]: a randomized, double-blind, multicenter Phase IIIa study (CAPTAIN; GSK 205715; NCT02924688), focused on a Japanese subpopulation, and a 52-week, open-label, Phase III long-term safety study conducted in Japanese patients with asthma (GSK 207236; NCT03184987).

Study designs have been described in detail elsewhere [13,14]. Briefly, in CAPTAIN, participants with uncontrolled asthma despite ICS/LABA were randomized 1:1:1:1:1:1 to receive once-daily treatment via the ELLIPTA inhaler with one of six regimens: FF/VI 100/25 mcg; FF/VI 200/25 mcg; FF/UMEC/VI 100/31.25/25 mcg (lower than the approved UMEC dose); FF/UMEC/VI 200/31.25/25 mcg (lower than the approved UMEC dose); FF/UMEC/VI 100/62.5/25 mcg; or FF/UMEC/VI 200/62.5/25 mcg for up to 52 weeks. In the long-term safety study, participants who were receiving maintenance therapy with ICS/LABA ± LAMA were allocated without randomization to once-daily FF/UMEC/VI via the ELLIPTA inhaler based on asthma control status during the run-in to either 100/62.5/25 mcg or 200/62.5/25 mcg for 52 weeks. Switching from 100/62.5/25 mcg to 200/62.5/25 mcg was permitted at Week 24 based on investigator-assessed asthma control.

### 2.3. Participants

Eligibility criteria for both studies have previously been published [13,14] and are as follows. CAPTAIN enrolled symptomatic adults aged ≥18 years with uncontrolled asthma despite ≥12 weeks of daily ICS/LABA maintenance prior to screening. Participants had no changes to therapy in the 6 weeks immediately prior to pre-screening, documented healthcare contact or temporary changes in asthma therapy for acute asthma symptoms within 1 year prior to screening, and an ACQ-6 total score ≥1.5 (at both screening and enrollment). Participants were also required to have a pre-bronchodilator morning trough forced expiratory volume in 1 s (FEV_1_) of ≥30–<85% of the predicted normal value at screening, and an increase in FEV_1_ of ≥12% and ≥200 mL 20–60 min following four salbutamol/albuterol inhalations at screening. Key exclusions included chronic obstructive pulmonary disease (COPD) or other respiratory disorders, including pneumonia, and current and former smokers with a ≥10 pack-year smoking history.

The long-term safety study enrolled Japanese participants aged ≥18 years with an asthma diagnosis ≥1-year prior to providing informed consent who were receiving maintenance therapy with ICS/LABA (±LAMA) for ≥4 weeks before screening. Participants were excluded if an asthma exacerbation occurred that required a change in maintenance therapy in the 6 weeks prior to screening (participants requiring a temporary change in asthma therapy were not necessarily excluded provided their condition had stabilized upon resuming pre-exacerbation maintenance), if they had a diagnosis of other concurrent respiratory disorders (e.g., COPD), were current smokers (within 12 months of screening), or former smokers with a smoking history of ≥10 pack-years.

### 2.4. Endpoints

Endpoints for both studies have previously been reported [13,14]. Briefly, while CAPTAIN evaluated lung function and exacerbation rates, the long-term safety study focused on safety and tolerability outcomes.

This post hoc analysis assessed CR at Week 24 and 52, which was not a pre-specified endpoint in either study. CR was assessed using three different definitions, which were adapted for use in this analysis (Table 1). The adapted Workgroup and JGL definitions are four-component composite definitions, while PGAM is a three-component composite definition. A participant was considered to have met the CR criteria (24 weeks) or have strong potential to achieve CR (52 weeks) at a given timepoint if they met all the criteria in a definition. Components of the three definitions used in this analysis are described below.

Briefly, the adapted Workgroup definition included the following four components:
  - SCS free;
  - Severe exacerbation free;
  - Asthma control defined as ACQ-5 total score <1.5;
  - Stabilized (change from baseline [CFB] in trough FEV_1_ ≥0 mL) and optimized (CFB in trough FEV_1_ ≥100 mL) lung function.
The adapted JGL definition included:
  - No SCS use;
  - Severe exacerbation free;
  - Asthma control defined as ACQ ≤0.75;
  - Stabilized (CFB in trough FEV_1_ ≥0 mL) and optimized (CFB in trough FEV_1_ ≥100 mL) lung function.
The adapted PGAM definition included:
  - No regular SCS use;
  - Severe exacerbation free;
  - Asthma control defined as ACQ ≤0.75.

### 2.5. Statistical Analysis

This was a post hoc, descriptive analysis, as the small sample size of both the Japanese subpopulation of the CAPTAIN trial and the long-term safety study was not sufficient for statistical analysis. The proportions of participants meeting the CR criteria at Week 24 and achieving CR at Week 52 were summarized. Descriptive analyses (summaries) were also performed to explore the impact of differing CFB in trough FEV_1_ thresholds (≥100 mL or ≥0 mL) on the proportion of patients meeting the CR criteria at Week 24 and achieving CR at Week 52 in both studies. In the CAPTAIN Japanese Subpopulation, only groups that include the approved UMEC dose (62.5 mcg) are presented, as well as the comparator FF/VI (4 of the 6 treatment groups): FF100/VI, FF100/UMEC/VI, FF200/VI, and FF200/UMEC/VI. In the long-term safety study, FF100/UMEC/VI, switched FF/UMEC/VI, and FF200/UMEC/VI groups are presented. Further statistical analyses were conducted on the intention-to-treat (ITT) population (as described elsewhere [12]).

## 3. Results

### 3.1. Participant Disposition and Baseline Characteristics

Of the overall ITT population (*N* = 2436) in the CAPTAIN study, 229 participants (9%) were Japanese, of whom 153 participants were randomized to either of the two FF/UMEC/VI treatment groups with the approved UMEC dose (62.5 mcg) or to either of the two FF/VI treatment groups and were included in this post hoc analysis [12,13]. Baseline characteristics were generally similar between the four treatment groups (Supplementary Table S1). The mean (standard deviation [SD]) age of Japanese participants was 54.0 (12.4) years, and 45% (*n* = 102) of the total Japanese participants were male. A total of 83% (*n* = 191) had ≥1 exacerbation in the 12 months prior to screening.

All participants from the long-term safety study (*N* = 111) were included in this post hoc analysis [14]. Baseline characteristics were similar between the three treatment groups (Supplementary Table S2). The mean (SD) age of participants was 50.9 (13.41) years, and 42% (*n* = 47) of participants were male. A total of 25% (*n* = 28) had ≥1 exacerbation in the 12 months prior to screening.

### 3.2. CR Assessment in the CAPTAIN Japanese Subpopulation

**Figure 1:**
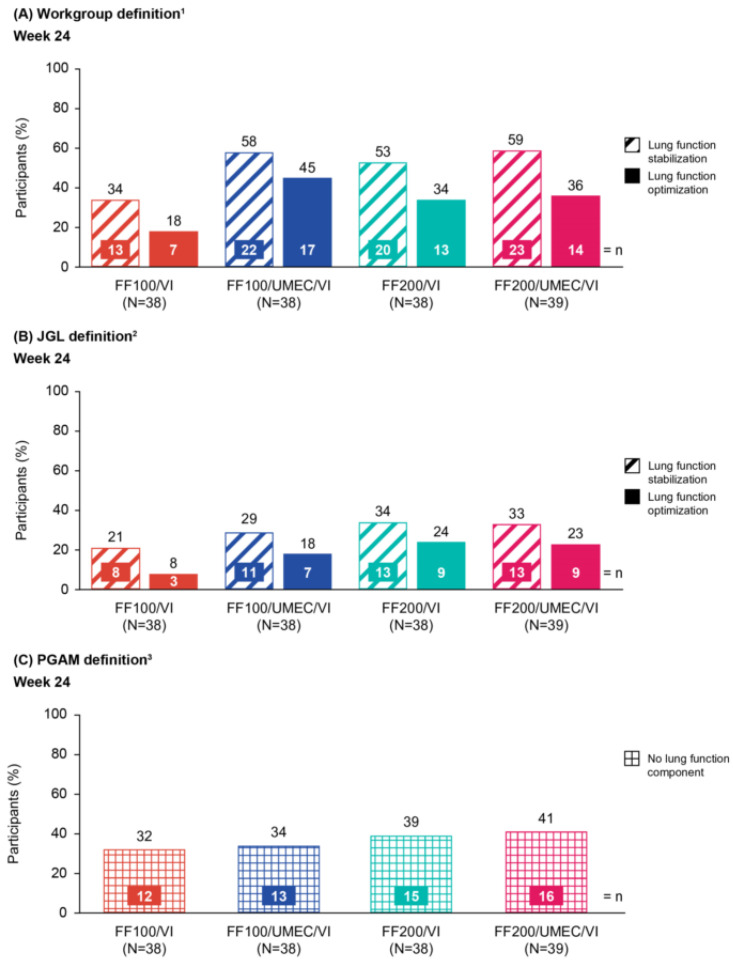
Proportion of participants meeting the CR criteria from the CAPTAIN Japanese subpopulation at Week 24 using (**A**) Workgroup definition, (**B**) JGL definition, and (**C**) PGAM definition. All doses are in mcg. ^1^ The Workgroup definition of CR was defined as: SCS-free, severe exacerbation-free, ACQ-5 total score <1.5, and a CFB in trough FEV_1_ of either ≥100 mL (optimized) or ≥0 mL (stabilized); ^2^ the JGL definition of CR was defined as: no SCS use, severe exacerbation-free, ACQ ≤0.75, and a CFB in trough FEV_1_ of either ≥100 mL (optimized) or ≥0 mL (stabilized); ^3^ the PGAM definition of CR was defined as: no regular SCS use, severe exacerbation-free, and ACQ ≤0.75. ACQ-5, Asthma Control Questionnaire 5-item; CFB, change from baseline; CR, clinical remission; FEV_1_, forced expiratory volume in 1 s; FF, fluticasone furoate; JGL, Japanese Guidelines for adult asthma; PGAM, Practical Guidelines for Asthma Management; SCS, systemic corticosteroid; UMEC, umeclidinium; VI, vilanterol.

Using the Workgroup definition, a higher proportion of participants in the CAPTAIN Japanese ITT subpopulation met the CR criteria at Week 24 with FF/UMEC/VI compared with FF/VI, irrespective of ICS dose and lung function threshold (Figure 1A). This was also the case for the overall ITT population [17]. When lung function was stabilized (CFB in trough FEV_1_ ≥0 mL), the criteria for CR were met in 34% (*n* = 13; FF100/VI), 58% (*n* = 22; FF100/UMEC/VI), 53% (*n* = 20; FF200/VI), and 59% (*n* = 23; FF200/UMEC/VI) of participants. Equivalent figures in the overall ITT population were 31% (*n* = 127), 42% (*n* = 170), 36% (*n* = 145), and 47% (*n* = 190), respectively [17]. When lung function was optimized (CFB in trough FEV_1_ ≥100 mL), the criteria for CR were met in 18% (*n* = 7; FF100/VI), 45% (*n* = 17; FF100/UMEC/VI), 34% (*n* = 13; FF200/VI), and 36% (*n* = 14; FF200/UMEC/VI) of participants. Equivalent figures in the overall ITT population were 19% (*n* = 77), 31% (*n* = 127), 26% (*n* = 104), and 36% (*n* = 146), respectively [17].

Baseline age, sex, body mass index (BMI), pre-bronchodilator FEV_1_, asthma duration, and age of disease onset were broadly similar between participants who met the CR criteria (*N* = 69) and participants who did not (*N* = 160), using the lung function optimization threshold (CFB in trough FEV_1_ ≥100 mL) (Supplementary Table S3). Participants who met the CR criteria had lower mean (SD) ACQ-5 total scores (1.68 [0.741]) and were less likely to have ≥2 exacerbations requiring SCS in the prior 12 months (12% [*n* = 8]) than participants who did not meet the CR criteria.

Using the JGL definition, 21% (*n* = 8; FF100/VI), 29% (*n* = 11; FF100/UMEC/VI), 34% (*n* = 13; FF200/VI), and 33% (*n* = 13; FF200/UMEC/VI) of participants met the CR criteria when using the stabilized lung function threshold, while lower proportions of participants met the CR criteria when using the optimized threshold (8% [*n* = 3], 18% [*n* = 7], 24% [*n* = 9], and 23% [*n* = 9], respectively) (Figure 1B).

Using the PGAM definition, which is less stringent than the JGL definition without the lung function component, 32% (*n* = 12) of participants (FF100/VI), 34% (*n* = 13; FF100/UMEC/VI), 39% (*n* = 15; FF200/VI), and 41% (*n* = 16; FF200/UMEC/VI) met the CR criteria (Figure 1C).

### 3.3. CR Assessment in the Long-Term Safety Study

**Figure 2:**
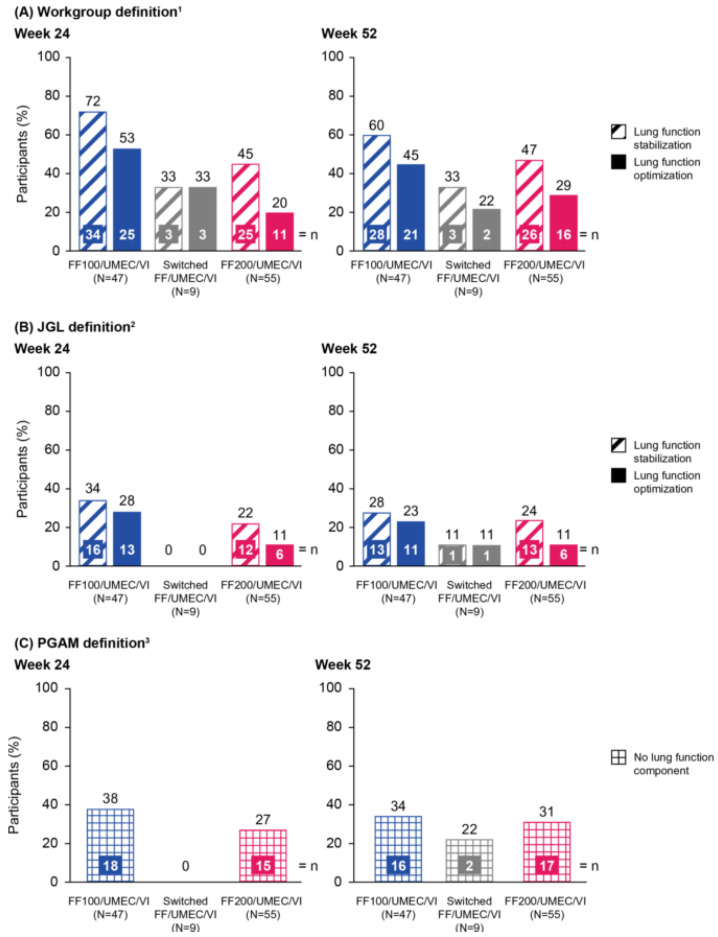
Proportion of participants meeting the CR criteria (Week 24) or achieving CR (Week 52) from the long-term safety study using (**A**) Workgroup definition, (**B**) JGL definition, and (**C**) PGAM definition. All doses are in mcg. ^1^ The Workgroup definition of CR was defined as: SCS-free, severe exacerbation-free, ACQ-5 total score <1.5, and a CFB in trough FEV_1_ of either ≥100 mL (optimized) or ≥0 mL (stabilized); ^2^ the JGL definition of CR was defined as: no SCS use, severe exacerbation-free, ACQ ≤0.75, and a CFB in trough FEV_1_ of either ≥100 mL (optimized) or ≥0 mL (stabilized); ^3^ the PGAM definition of CR was defined as: no regular SCS use, severe exacerbation-free, and ACQ ≤0.75. ACQ-5, Asthma Control Questionnaire 5-item; CFB, change from baseline; CR, clinical remission; FEV_1_, forced expiratory volume in 1 s; FF, fluticasone furoate; JGL, Japanese Guidelines for adult asthma; PGAM, Practical Guidelines for Asthma Management; SCS, systemic corticosteroid; UMEC, umeclidinium; VI, vilanterol.

In the long-term safety study, using the Workgroup definition, 22–60% of participants achieved CR at Week 52 across treatment groups, irrespective of the lung function threshold assessed (Figure 2A). Using the stabilized lung function threshold, more participants achieved CR (FF100/UMEC/VI: 60% [*n* = 28]; switched FF/UMEC/VI: 33% [*n* = 3]; and FF200/UMEC/VI: 47% [*n* = 26]) than when using the optimized threshold (FF100/UMEC/VI: 45% [*n* = 21]; switched FF/UMEC/VI: 22% [*n* = 2]; and FF200/UMEC/VI: 29% [*n* = 16]). At Week 24, similar proportions of participants met the CR criteria in the FF100/UMEC/VI and FF200/UMEC/VI groups.

Baseline age, sex, BMI, ACQ-5 total scores, and pre-bronchodilator FEV_1_ were broadly similar between participants who achieved CR (*N* = 39) and those who did not (*N* = 72) at Week 52, using the lung function optimization threshold (CFB in trough FEV_1_ ≥100 mL) (Supplementary Table S4). Participants who achieved CR had a longer mean (SD) asthma duration (22.0 [14.2] years) and earlier mean (SD) disease onset (28.4 [18.9] years), and were more likely to have been free of SCS-treated exacerbations in the prior 12 months (79%, *n* = 31) than participants who did not achieve CR. No participants who achieved CR experienced ≥2 exacerbations.

At Week 52 using the JGL definition, 28% (*n* = 13) of participants (FF100/UMEC/VI), 11% (*n* = 1; switched FF/UMEC/VI), and 24% (*n* = 13; FF200/UMEC/VI) achieved CR using the stabilized lung function threshold, and 23% (*n* = 11; FF100/UMEC/VI), and 11% (*n* = 1; switched FF/UMEC/VI and *n* = 6; FF200/UMEC/VI), achieved CR using the optimized lung function threshold. At Week 24, similar proportions of participants met the CR criteria in the FF100/UMEC/VI and F200/UMEC/VI groups (Figure 2B).

Using the PGAM definition, 34% (*n* = 16) of participants (FF100/UMEC/VI), 22% (*n* = 2; switched FF/UMEC/VI), and 31% (*n* = 17; FF200/UMEC/VI) achieved CR at Week 52. Similar results were observed at Week 24 in the FF100/UMEC/VI and FF200/UMEC/VI groups (Figure 2C).

### 3.4. Impact of Number of Components on CR Attainability

In both studies, the number of participants who met the CR criteria (Week 24) or achieved CR (Week 52) decreased with increasing number of components assessed, with the fewest participants attaining CR when the lung function component (stabilized and optimized) was included.

**Figure 3:**
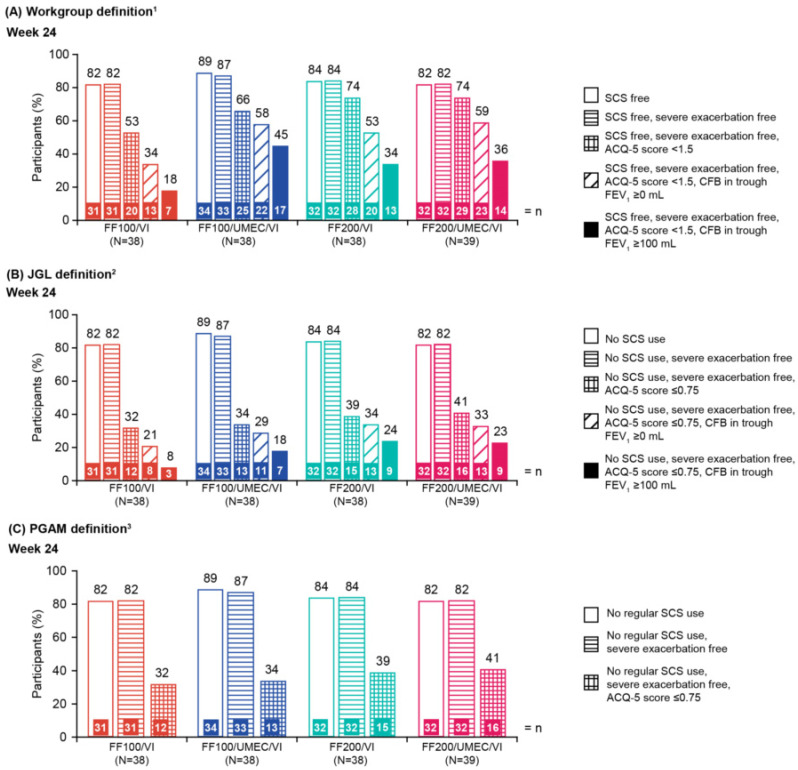
Impact of each component of the CR definition on the proportion of participants meeting the CR criteria from the CAPTAIN Japanese subpopulation at Week 24 using (**A**) Workgroup definition, (**B**) JGL definition, and (**C**) PGAM definition. All doses are in mcg. ^1^ The Workgroup definition of CR was defined as: SCS-free, severe exacerbation-free, ACQ-5 total score <1.5, and a CFB in trough FEV_1_ of either ≥100 mL (optimized) or ≥0 mL (stabilized); ^2^ the JGL definition of CR was defined as: no SCS use, severe exacerbation-free, ACQ ≤0.75, and a CFB in trough FEV_1_ of either ≥100 mL (optimized) or ≥0 mL (stabilized); ^3^ the PGAM definition of CR was defined as: no regular SCS use, severe exacerbation-free, and ACQ ≤0.75. ACQ-5, Asthma Control Questionnaire 5-item; CFB, change from baseline; CR, clinical remission; FEV_1_, forced expiratory volume in 1 s; FF, fluticasone furoate; JGL, Japanese Guidelines for adult asthma; PGAM, Practical Guidelines for Asthma Management; SCS, systemic corticosteroid; UMEC, umeclidinium; VI, vilanterol.

At Week 24 in the CAPTAIN Japanese ITT subpopulation, using the Workgroup definition, the proportion of participants who were SCS-free was 82% (FF100/VI), 89% (FF100/UMEC/VI), 84% (FF200/VI), and 82% (FF200/UMEC/VI) (Figure 3A). Similar proportions of participants met the CR criteria of SCS-free + severe exacerbation-free. The proportion of participants who met the CR criteria of SCS-free + severe exacerbation-free + ACQ-5 score <1.5 were 53% (FF100/VI), 66% (FF100/UMEC/VI), 74% (FF200/VI), and 74% (FF200/UMEC/VI), and when the stabilized lung function threshold was added, those who met the criteria decreased to 34%, 58%, 53%, and 59%, respectively. Equivalent figures were 18%, 45%, 34%, and 36%, respectively, when the optimized lung function threshold was used.

The trend was similar using the JGL or PGAM definitions. As expected, lower proportions of participants met the CR criteria of no SCS use + severe exacerbation free + ACQ-5 score ≤0.75, as it is more stringent (32% [FF100/VI], 34% [FF100/UMEC/VI], 39% [FF200/VI], and 41% [FF200/UMEC/VI]). Those who met the criteria decreased to 21%, 29%, 34%, and 33%, respectively, when the stabilized lung function threshold was used, and to 8%, 18%, 24%, and 23%, respectively, when the optimized lung function threshold was used (Figure 3B,C).

**Figure 4:**
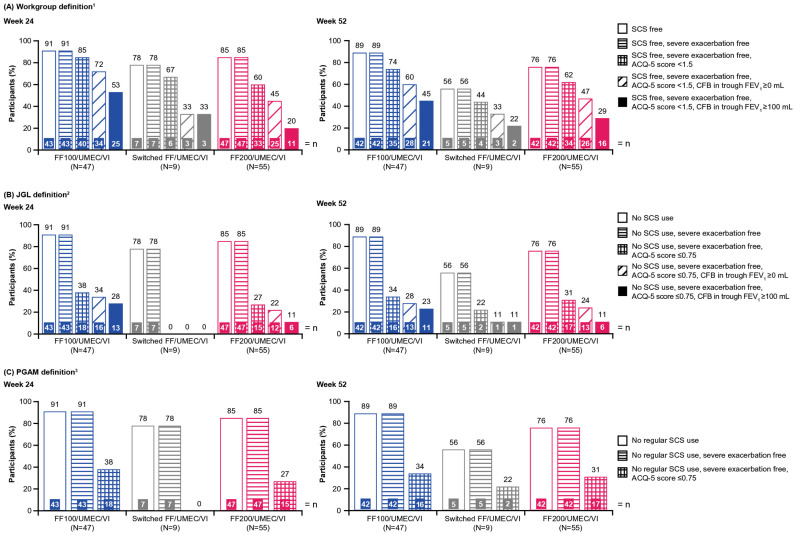
Impact of each component of the CR definition on the proportion of participants meeting the CR criteria (Week 24) or achieving CR (Week 52) from the long-term safety study using (**A**) the Workgroup definition, (**B**) JGL definition, and (**C**) PGAM definition. All doses are in mcg. ^1^ The Workgroup definition of CR was defined as: SCS-free, severe exacerbation-free, ACQ-5 total score <1.5, and a CFB in trough FEV_1_ of either ≥100 mL (optimized) or ≥0 mL (stabilized); ^2^ the JGL definition of CR was defined as: no SCS use, severe exacerbation-free, ACQ ≤0.75, and a CFB in trough FEV_1_ of either ≥100 mL (optimized) or ≥0 mL (stabilized); ^3^ the PGAM definition of CR was defined as: no regular SCS use, severe exacerbation-free, and ACQ ≤0.75. ACQ-5, Asthma Control Questionnaire 5-item; CFB, change from baseline; CR, clinical remission; FEV_1_, forced expiratory volume in 1 s; FF, fluticasone furoate; JGL, Japanese Guidelines for adult asthma; PGAM, Practical Guidelines for Asthma Management; SCS, systemic corticosteroid; UMEC, umeclidinium; VI, vilanterol.

At Week 52 in the long-term safety study, using the Workgroup definition, the proportion of participants who achieved CR criteria of SCS-free were 89% (FF100/UMEC/VI), 56% (switched FF/UMEC/VI), and 76% (FF200/UMEC/VI); and SCS-free + severe exacerbation-free were 89%, 56%, and 76%, respectively (Figure 4A). The proportion of participants who achieved CR criteria of SCS-free + severe exacerbation-free + ACQ-5 score <1.5 was 74% (FF100/UMEC/VI), 44% (switched FF/UMEC/VI), and 62% (FF200/UMEC/VI), and when the stabilized lung function threshold was added, those who met the criteria decreased to 60%, 33%, and 47%, respectively. Equivalent figures were 45%, 22%, and 29% when the optimized lung function threshold was used. Similar trends were reported at Week 24.

The trend was similar using the PGAM or JGL definitions. The proportion of participants who achieved CR criteria of no SCS use + severe exacerbation-free + ACQ-5 score ≤0.75 was 34% (FF100/UMEC/VI), 22% (switched FF100/UMEC/VI), and 31% (FF200/UMEC/VI), and when the stabilized lung function threshold was added, those who achieved the criteria decreased to 28%, 11%, and 24%, respectively. Equivalent figures were 23%, 11%, and 11% when the optimized lung function threshold was used. Similar trends were reported at Week 24 (Figure 4B,C).

Assessment of the attainability of individual components of CR can be found in the Supplementary Results S1.

## 4. Discussion

This post hoc analysis of the Japanese subpopulation of the CAPTAIN study and the long-term safety study demonstrated that CR is attainable with inhaled FF/UMEC/VI in participants from CAPTAIN who have uncontrolled asthma despite ICS/LABA and Japanese patients with moderate-to-severe asthma previously on ICS/LABA ± LAMA therapy. Results from the Japanese ITT subpopulation of the CAPTAIN study were aligned with those from the overall CAPTAIN ITT population [17]. This suggests that Japanese participants respond to triple therapy similarly to the overall population in terms of CR attainability.

CR attainability in this patient population was most influenced by changes in symptom control and lung function criteria, with more stringent cut-offs resulting in fewer participants attaining each criterion. The Workgroup definition (ACQ-5 total score <1.5 and CFB in trough FEV_1_ ≥0 mL) identified the greatest proportion of participants achieving CR across all studies and time points analyzed, while the JGL definition with more stringent criteria (ACQ-5 total score ≤0.75 and CFB in trough FEV_1_ ≥100 mL) identified the fewest. Component-level analysis revealed that lung function was the main barrier to achieving full CR under JGL criteria. Despite differences in study designs [13,14], this trend was observed across both studies in this analysis. Interestingly, data at Week 24 in both studies showed similar trends, and although data from the CAPTAIN Japanese ITT subpopulation were only assessed at Week 24, due to the small sample size at Week 52, it is anticipated that these data will help inform future CR research.

The choice of symptom control measures impacts CR attainability. It is not clear from this study whether JGL and PGAM definitions (ACQ-5 total score ≤0.75) or the Workgroup definition (ACQ-5 total score <1.5) are more appropriate when assessing CR, as no long-term observations have been made [6,7,8]. The present analysis used the ACQ-5 total score because it was prospectively collected in both trials. However, the PGAM definition assesses symptom control using the ACT score, typically requiring an ACT ≥23 [7,16]. JGL allows use of either ACT ≥23 or ACQ-5 total score ≤0.75 for the evaluation of symptom control [6]. ACQ-5 and ACT measure overlapping but not identical aspects of asthma control, and their cut-points are not interchangeable. As such, further research is required to confirm the validity of these components based on post-CR achievement observations. In clinical settings, expectations around asthma treatment should include asthma control and stabilized or optimized lung function as well as elimination of exacerbations and SCS use, which comprise the components of CR.

There were no major differences in baseline characteristics between participants who met or achieved CR across studies, but participants who did not meet or achieve CR were more likely to have recent exacerbations in both studies, higher mean ACQ-5 total scores in the CAPTAIN Japanese ITT subpopulation, and shorter disease duration in the long-term safety study. Interestingly, post hoc analyses of clinical trials of patients with severe asthma being treated with biologics have reported that predictors of remission include the presence of a shorter duration of illness, younger age, preserved lung function at initiation of biological therapy, and either lower or no maintenance SCS [18]. While shorter disease duration appears not to be a predictor of CR in this analysis, which may be related to the low sample size, as is the case for biologics, careful monitoring and appropriate treatment choices may be necessary for patients with asthma on inhaled therapies early in the treatment paradigm.

A systematic review and meta-analysis of CR in patients with severe asthma treated with biologics reported CR rates ranging from 18% to 69% in 25 studies [19], while post hoc analyses of clinical trials of participants being treated with biologics have reported CR rates ranging from 15% to 38% [16]. The present data overlap with what is seen in participants receiving biologics and range from 0% to 72% overall for Japanese patients with moderate-to-severe asthma receiving inhaled therapies. As well as differences due to the different patient populations studied in these trials, it is important to note that alternate definitions for asthma control (i.e., ACQ-5 total score <1.5 or ACT ≥20) and lung function (i.e., post-bronchodilator FEV_1_ ≥80% predicted or pre-bronchodilator FEV_1_ ≥100 mL) limit comparison of these results [18].

A value of this analysis is that it is one of the few to evaluate CR in Japanese patients with moderate-to-severe asthma. Furthermore, this study has demonstrated concordant results across two robust Phase III clinical trials. Despite this, the open-label design of the long-term safety study and post hoc nature of this analysis mean that results are descriptive and exploratory in nature and may not fully reflect real-world settings. The small sample size of both studies prevented statistical analysis. Small participant numbers in the CAPTAIN Japanese subpopulation at Week 52 were prohibitive and meant that only CR at Week 24 data are reported. Particular caution should be taken when interpreting data from participants in the long-term safety study who stepped up from FF/UMEC/VI 100/62.5/25 to 200/62.5/25 mcg, as there were only 9 participants in this group. The lack of comorbidities data in this analysis limited the evaluation of their importance by CR status; of note, the presence of particular comorbidities has previously been identified as a barrier to meeting/achieving CR in patients with severe asthma treated with biologics [19] and may be of interest in future studies. The lack of ACT data in this analysis limited the evaluation of CR according to the JGL and PGAM definitions. Furthermore, as there were no long-term observations made in this study, maintenance of ACT ≥23 for >1 year, as suggested by JGL to monitor asthma symptom control maintenance, was not assessed. It is also important to note that the definition of OCS use in Japan, in the context of CR, differs from definitions in other countries, as it refers specifically to OCS prescribed as maintenance therapy, i.e., OCS that has been used for more than one month. This may lead to an underestimation of the proportion of patients meeting the SCS component, as the definitions used in this analysis are more stringent.

## 5. Conclusions

CR is an attainable treatment goal in Japanese patients with moderate-to-severe asthma treated with the single-inhaler triple therapy FF/UMEC/VI, not yet eligible for biologics. This study shows that the attainability of CR varies depending on the definition used; further consideration of the most appropriate definition is required. Nonetheless, the findings of this study support the integration of CR into treatment expectations for appropriate patients in clinical practice.

## Figures and Tables

**Table 1:** CR definitions used in this analysis.

	Workgroup		JGL		PGAM	
	Original Definition ^1^	Adapted Definition Used in This Analysis	Original Definition	Adapted Definition Used in This Analysis	Original Definition	Adapted Definition Used in This Analysis
SCS use	SCS free (no recorded oral or injectable corticosteroids)	SCS free (no recorded oral, intravenous, or subcutaneous corticosteroids), as the original definition	No long-term regular OCS use ^2^	No SCS use (no recorded oral, intravenous, or subcutaneous corticosteroids)	No regular OCS use	No regular SCS use (no recorded oral, intravenous, or subcutaneous corticosteroids)
Exacerbation history	No exacerbations (requiring a physician visit, emergency care, hospitalization, and/or SCS for asthma [i.e., oral, injectable])	Severe exacerbation free (requiring hospital admission or ER visit due to the need for SCS, or an asthma deterioration requiring SCS use [or doubling of the current maintenance SCS dose] for ≥3 days)	No exacerbations (requiring SCS)	Severe exacerbation free (requiring SCS), as the original definition	No exacerbations (requiring SCS, ER visits, or hospitalization)	Severe exacerbation free (requiring SCS, ER visits, or hospitalization), as the original definition
Asthma control	ACT >20, AirQ <2, ACQ <0.75	ACQ-5 total score <1.5	ACQ ≤0.75, ACT ≥23	ACQ ≤0.75	ACT ≥23	ACQ ≤0.75
Lung function	Stabilized Optimized	Stabilized (CFB in trough FEV_1_ ≥0 mL) Optimized (CFB in trough FEV_1_ ≥100 mL)	Stabilized (% predicted FEV_1_ change <10%, decline in FEV_1_ over time of 30 mL/year, or PEF daily change <10%) Normalized (% predicted FEV_1_ ≥80%)	Stabilized (CFB in trough FEV_1_ ≥0 mL) Optimized (CFB in trough FEV_1_ ≥100 mL)	Not included in the definition	N/A

^1^ The complete CR criteria outlined by the Workgroup consensus statement also include no missed work/school; continued use of controller therapies at low–medium ICS dose; and rescue therapy use no more than once per month [8], ^2^ including use for diseases other than asthma. ACQ, Asthma Control Questionnaire; ACT, Asthma Control Test; AirQ, Asthma Impairment and Risk Questionnaire; CFB, change from baseline; CR, clinical remission; ER, emergency room; FEV_1_, forced expiratory volume in 1 s; ICS, inhaled corticosteroid; JGL, Japanese Guidelines for adult asthma; N/A, not applicable; OCS, oral corticosteroid; PEF, peak expiratory flow; PGAM, Practical Guidelines for Asthma Management; SCS, systemic corticosteroid.

## Data Availability

Please refer to the GSK weblink to access GSK’s data sharing policies and, as applicable, seek anonymized subject-level data via the link https://www.gsk-studyregister.com/en/.

## Supplementary Materials

The following supporting information can be downloaded at: https://www.mdpi.com/article/10.3390/jcm15041497/s1, Supplementary Results S1: Individual Component Drivers of CR Attainment; Supplementary Table S1: Baseline characteristics for the CAPTAIN Japanese subpopulation [13]; Supplementary Table S2: Baseline characteristics for the long-term safety study [14]; Supplementary Table S3: Baseline participant characteristics by CR status at Week 24 (CAPTAIN Japanese subpopulation); Supplementary Table S4: Baseline participant characteristics by CR status at Week 52 (long-term safety study); Supplementary Table S5: Proportion of participants meeting CR criteria at Week 24 by individual component (non-cumulative) (CAPTAIN Japanese subpopulation); Supplementary Table S6: Proportion of participants meeting the CR criteria (Week 24) or achieving CR (Week 52) by individual component (non-cumulative) (long-term safety study).

## Abbreviations

ACQ Asthma Control Questionnaire; ACT, Asthma Control Test; AirQ, Asthma Impairment and Risk Questionnaire; BMI, body mass index; CFB, change from baseline; COPD, chronic obstructive pulmonary disease; CR, clinical remission; ER, emergency room; FEV_1_, forced expiratory volume in 1 s; FF, fluticasone furoate; ICS, inhaled corticosteroid; ITT, intention-to-treat; JGL, Japanese Guidelines for adult asthma; LABA, long-acting β_2_-agonist; LAMA, long-acting muscarinic antagonist; N/A, not applicable; OCS, oral corticosteroid; PGAM, Practical Guidelines for Asthma Management; PEF, peak expiratory flow; SCS, systemic corticosteroid; SD, standard deviation; UMEC, umeclidinium; US, United States; VI, vilanterol.
