# Supplementary material for: Is Clinical Remission, an Ambitious Treatment Goal, Achievable in Patients with Moderate-to-Severe Asthma on Inhaled Therapies: How Ambitious Should We Be?
Source: J Clin Med. 2026 Feb 14;15(4):1497. doi: 10.3390/jcm15041497 (PMC12941637; doi:10.3390/jcm15041497)
Supplement: Supplementary file 1 [file jcm-15-01497-s001.zip › jcm-4065727-supplementary.pdf]

Supplementary materials

Supplementary Results S1

Individual Component Drivers of CR Attainment

For both studies, while not all CR criteria were met, a substantial proportion of participants achieved individual components of CR.

At Week 24 in the CAPTAIN Japanese ITT subpopulation, more than 80% of participants in each treatment group were SCS free (FF100/VI 82% [n=31], FF100/UMEC/VI 89% [n=34], FF200/VI 84% [n=32], and FF200/UMEC/VI 82% [n=32]) or severe exacerbation free (FF100/VI 87% [n=33], FF100/UMEC/VI 92% [n=35], FF200/VI 89% [n=34], and FF200/UMEC/VI 95% [n=37]) (**Supplementary Table S5**). Using the ACQ-5 total score <1.5 criterion from the Workgroup definition, 61% (n=23) of participants in the FF100/VI group, 71% (n=27; FF100/UMEC/VI), 82% (n=31; FF200/VI), and 87% (n=34; FF200/UMEC/VI) met this component , and 37% (n=14; FF100/VI), 34% (n=13; FF100/UMEC/VI), 45% (n=17; FF200/VI), and 49% (n=19; FF200/UMEC/VI) met this component when using the ACQ-5 total score ≤0.75 cut-off. A total of 58% (n=22) of participants in the FF100/VI group, 74% (n=28; FF100/UMEC/VI), 66% (n=25; FF200/VI), and 74% (n=29; FF200/UMEC/VI) had a CFB in trough FEV_1 ≥0 mL and equivalent figures were 26%, 55%, 39%, and 49% when using a CFB in trough FEV_1 ≥100 mL.

At Week 52 in the long-term safety study, over 55% of the participants across all treatment groups were SCS free or severe exacerbation free (FF100/UMEC/VI 89% [n=42], switched FF/UMEC/VI 56% [n=5], for both components; and FF200/UMEC/VI 76% [n=42] and 80% [n=44], respectively) (**Supplementary Table S6**). A total of 83% (n=39) of participants in the FF100/UMEC/VI group, 67% (n=6; switched FF/UMEC/VI), and 64% (n=35; FF200/UMEC/VI) achieved the Workgroup threshold of ACQ-5 total score <1.5. Fewer participants met the more stringent JGL/PGAM cut-off of ≤0.75 (43% [n=20]; FF100/UMEC/VI; 0%; switched FF/UMEC/VI; and 29% [n=16; FF200/UMEC/VI). A total of 77% (n=36) of participants in the FF100/UMEC/VI group, 56% (n=5; switched FF100/UMEC/VI), and 69% (n=38; FF200/UMEC/VI) had a CFB in trough FEV_1 ≥0 mL and

equivalent figures were 60%, 33%, and 42% when using a CFB in trough FEV_1 ≥100 mL. Similar trends were seen at Week 24.

Tables

Supplementary Table S1. Baseline characteristics for the CAPTAIN Japanese subpopulation [13]

	**FF100/VI (N=38)**	**FF100/UMEC/VI (N=38)**	**FF200/VI (N=38)**	**FF200/UMEC/VI (N=39)**	**Total (N=229)**[1]
**Age, years**[2], mean (SD)	56 (12.6)	54 (11.7)	53 (11.7)	57 (12.4)	54 (12.4)
**Male,** n (%)	18 (47)	18 (47)	18 (47)	19 (49)	102 (45)
**BMI, kg/m^2,** mean (SD)	25.6 (5.0)	23.6 (3.9)	25.6 (3.4)	24.8 (3.6)	25.1 (4.4)
**Former smoker,** n (%)	6 (16)	12 (32)	8 (21)	14 (36)	59 (26)
**ACQ-6 total score at randomization,** mean (SD)[3]	1.702 (0.657)	1.509 (0.754)	1.548 (0.691)	1.624 (0.710)	1.614 (0.680)
**% predicted FEV$_1$, pre-bronchodilator,** mean (SD)	74.9 (12.7)	74.4 (14.5)	75.3 (14.1)	71.1 (16.0)	71.7 (15.0)
**Exacerbations in prior year,** n (%)					
**1 moderate/severe**	15 (39)	18 (47)	21 (55)	19 (49)	103 (45)
**≥2 severe**	15 (39)	16 (42)	9 (24)	17 (44)	88 (38)
**Pre-study ICS dose,** n (%)					
**Medium dose**	26 (68)	26 (68)	26 (68)	25 (64)	156 (68)
**High dose**	12 (32)	12 (32)	12 (32)	14 (36)	73 (32)

All doses are in mcg. [1]Includes all Japanese participants, including those taking UMEC 31.25 mcg; [2]only year of birth was collected; age was derived at the date of the pre-screening visit and day and month of birth were imputed as June 30; [3]analysis of ACQ-6 total score at randomization was performed post hoc.

ACQ-6, Asthma Control Questionnaire 6-item; BMI, body mass index; FEV$_1$, forced expiratory volume in 1 s; FF, fluticasone furoate; ICS, inhaled corticosteroid; SD, standard deviation; UMEC, umeclidinium; VI, vilanterol.

Supplementary Table S2. Baseline characteristics for the long-term safety study [14]

	FF100/UMEC/VI (N=47)	Switched FF/UMEC/VI (N=9)	FF200/UMEC/VI (N=55)	Total (N=111)
**Age[1], years,** mean (SD)	48.0 (14.28)	48.4 (7.76)	53.8 (12.91)	50.9 (13.41)
**Male,** n (%)	24 (51)	4 (44)	19 (35)	47 (42)
**BMI, kg/m^2,** mean (SD)	23.8 (4.23)	24.3 (5.75)	24.4 (5.38)	24.2 (4.92)
**Former smoker,** n (%)	9 (19)	3 (33)	14 (25)	26 (23)
**ACQ-6 total score at randomization,** mean (SD)	1.4 (0.45)	1.5 (0.63)	1.3 (0.59)	1.4 (0.54)
**% predicted FEV$_1$,** mean (SD)	87.7 (14.77)	80.5 (14.82)	86.3 (18.29)	86.4 (16.58)
**Asthma duration, years,** mean (SD)	17.3 (14.08)	19.6 (15.01)	18.5 (14.37)	18.5 (14.37)
**Exacerbations requiring OCS[2],** n (%)				
**1 severe**	5 (11)	2 (22)	12 (22)	19 (17)
**≥2 severe**	3 (6)	1 (11)	5 (9)	9 (8)

All doses are in mcg. [1]Only year of birth was collected; age was derived at the date of the pre-screening visit and day and month of birth were imputed as June 30; [2]asthma exacerbations reported in the 12 months prior to the screening visit that required only OCS and did not involve hospitalization.

ACQ-6, Asthma Control Questionnaire 6-item; BMI, body mass index; FEV$_1$, forced expiratory volume in 1 s; FF, fluticasone furoate; OCS, oral corticosteroid; SD, standard deviation; UMEC, umeclidinium; VI, vilanterol.

Supplementary Table S3. Baseline participant characteristics by CR status at Week 24 (CAPTAIN Japanese subpopulation)

	Met the CR criteria					Did not meet the CR criteria				
	**FF100/VI (N=7)**	**FF100/UMEC/VI (N=17)**	**FF200/VI (N=13)**	**FF200/UMEC/VI (N=14)**	**Total (N=69)[1]**	**FF100/VI (N=31)**	**FF100/UMEC/VI (N=21)**	**FF200/VI (N=25)**	**FF200/UMEC/VI (N=25)**	**Total (N=160)[1]**
**Age[2], years,** mean (SD)	55.3 (8.22)	52.9 (13.98)	54.0 (14.42)	56.1 (13.74)	53.7 (13.15)	56.0 (13.45)	54.5 (9.77)	53.0 (10.30)	57.7 (11.88)	54.5 (12.13)
**Male,** n (%)	3 (43)	9 (53)	4 (31)	6 (43)	27 (39)	15 (48)	9 (43)	14 (56)	13 (52)	75 (47)
**BMI, kg/m^2,** mean (SD)	24.84 (2.184)	22.22 (3.236)	25.57 (3.759)	23.77 (2.839)	24.20 (3.959)	25.71 (5.430)	24.68 (4.154)	25.61 (3.347)	25.40 (3.850)	25.52 (4.483)
**ACQ-5 total score,** mean (SD)	1.60 (0.622)	1.39 (0.669)	1.92 (0.843)	1.84 (0.793)	1.68 (0.741)	2.00 (0.755)	2.01 (0.977)	1.74 (0.774)	1.84 (0.839)	1.93 (0.790)
**Baseline FEV$_1$, L,** mean (SD)	1.994 (0.7835)	2.001 (0.4530)	1.976 (0.9793)	1.821 (0.6408)	1.899 (0.6611)	2.075 (0.6825)	2.033 (0.6162)	2.162 (0.5920)	1.955 (0.5799)	2.033 (0.6182)
**Asthma duration, years,** mean (SD)	20.14 (16.365)	29.06 (18.866)	19.08 (15.036)	21.00 (20.081)	22.43 (16.589)	22.32 (14.689)	22.48 (16.609)	27.36 (14.227)	25.52 (17.386)	24.08 (15.171)
**Onset age of asthma, years,** mean (SD)	35.1 (17.82)	23.8 (18.10)	34.9 (17.11)	35.1 (22.98)	31.3 (18.87)	33.7 (24.94)	32.0 (17.51)	25.6 (16.74)	32.2 (16.69)	30.4 (19.77)
**Exacerbations requiring SCS,** n (%)[3]										
**0**	3 (43)	5 (29)	7 (54)	5 (36)	30 (43)	13 (42)	6 (29)	15 (60)	9 (36)	68 (43)
**1**	4 (57)	8 (47)	5 (38)	6 (43)	31 (45)	12 (39)	6 (29)	7 (28)	6 (24)	46 (29)
**≥2**	0	4 (24)	1 (8)	3 (21)	8 (12)	6 (19)	9 (43)	3 (12)	10 (40)	46 (29)

All doses are in mcg. [1]Includes all Japanese participants, including those taking UMEC 31.25 mcg; [2]only year of birth was collected; age was derived at the date of the pre-screening visit and day and month of birth were imputed as June 30; [3]asthma exacerbations reported in the 12 months prior to the screening visit that required only SCS and did not involve hospitalization.

ACQ-5, Asthma Control Questionnaire 5-item; BMI, body mass index; CR, clinical remission; FEV_1, forced expiratory volume in 1 s; FF, fluticasone furoate; SCS, systemic corticosteroid; SD, standard deviation; UMEC, umeclidinium; VI, vilanterol.

Supplementary Table S4. Baseline participant characteristics by CR status at Week 52 (long-term safety study)

	Achieved CR				Did not achieve CR			
	**FF100/UMEC/ VI (N=21)**	**Switched FF/UMEC/VI (N=2)**	**FF200/UMEC/ VI (N=16)**	**Total (N=39)**	**FF100/UMEC/ VI (N=26)**	**Switched FF/UMEC/VI (N=7)**	**FF200/UMEC/ VI (N=39)**	**Total (N=72)**
**Age[1], years,** mean (SD)	48.6 (13.65)	44.5 (4.95)	53.4 (11.14)	50.3 (12.46)	47.5 (15.02)	49.6 (8.34)	54.0 (13.7)	51.2 (13.97)
**Male,** n (%)	11 (52)	2 (100)	8 (50)	21 (54)	13 (50)	2 (29)	11 (28)	26 (36)
**BMI, kg/m^2,** mean (SD)	25.39 (3.804)	24.17 (3.864)	25.85 (4.721)	25.52 (4.118)	22.57 (4.204)	24.31 (6.444)	23.85 (5.575)	23.43 (5.178)
**ACQ-5,** mean (SD)	1.58 (0.469)	2.3 (0.707)	1.7 (0.649)	1.67 (0.566)	1.62 (0.526)	1.69 (0.855)	1.55 (0.709)	1.59 (0.656)
**Baseline FEV$_1$,** mean (SD)	2.415 (0.7796)	2.61 (0.5233)	2.182 (0.7026)	2.329 (0.7344)	2.469 (0.6671)	2.15 (0.7213)	2.285 (0.7281)	2.338 (0.7041)
**Asthma duration, years,** mean (SD)	20.43 (13.94)	26.5 (10.61)	23.38 (15.453)	21.95 (14.237)	14.69 (13.94)	15.29 (12.72)	18.08 (14.742)	16.58 (14.182)
**Onset age of asthma, years,** mean (SD)	28.1 (20.32)	18 (15.56)	30 (17.84)	28.4 (18.87)	32.8 (18.11)	34.3 (17.81)	35.9 (19.42)	34.7 (18.61)
**Exacerbations requiring SCS,** n (%)[2]								
**0**	18 (86)	2 (100)	11 (69)	31 (79)	21 (81)	4 (57)	27 (69)	52 (72)
**1**	3 (14)	0 (0)	5 (31)	8 (21)	2 (8)	2 (29)	7 (18)	11 (15)
**≥2**	0 (0)	0 (0)	0 (0)	0 (0)	3 (12)	1 (14)	5 (13)	9 (13)

All doses are in mcg. [1]Only year of birth was collected; age was derived at the date of the pre-screening visit and day and month of birth were imputed as June 30; [2]asthma exacerbations reported in the 12 months prior to the screening visit that required only SCS and did not involve hospitalization.

ACQ-5, Asthma Control Questionnaire 5-item; BMI, body mass index; CR, clinical remission; FEV_1, forced expiratory volume in 1 s; FF, fluticasone furoate; SCS, systemic corticosteroid; SD, standard deviation; UMEC, umeclidinium; VI, vilanterol.

Supplementary Table S5. Proportion of participants meeting CR criteria at Week 24 by individual component (non-cumulative) (CAPTAIN Japanese subpopulation)

**Patients,** n (%)	**FF100/VI (N=38)**	**FF100/UMEC/VI (N=38)**	**FF200/VI (N=38)**	**FF200/UMEC/VI (N=39)**
**Japanese subpopulation**				
**SCS free[1,2,3]**	31 (82)	34 (89)	32 (84)	32 (82)
**Severe exacerbation free[1,2,3]**	33 (87)	35 (92)	34 (89)	37 (95)
**ACQ-5 total score <1.5[1]**	23 (61)	27 (71)	31 (82)	34 (87)
**ACQ-5 total score ≤0.75[2,3]**	14 (37)	13 (34)	17 (45)	19 (49)
**CFB in trough FEV_1 ≥100 mL[1,2]**	10 (26)	21 (55)	15 (39)	19 (49)
**CFB in trough FEV_1 ≥0 mL[1,2]**	22 (58)	28 (74)	25 (66)	29 (74)
**Overall ITT population**				
**SCS free[1,2,3]**	324 (80)	338 (83)	340 (84)	342 (84)
**Severe exacerbation free[1,2,3]**	327 (80)	340 (84)	348 (86)	353 (87)
**ACQ-5 total score <1.5[1]**	240 (59)	263 (65)	259 (64)	268 (66)
**ACQ-5 total score ≤0.75[2,3]**	129 (32)	145 (36)	139 (34)	151 (37)
**CFB in trough FEV_1 ≥100 mL[1,2]**	129 (32)	200 (49)	151 (37)	210 (51)
**CFB in trough FEV_1 ≥0 mL[1,2]**	212 (52)	270 (67)	226 (56)	280 (69)

All doses are in mcg. [1]Components of the Workgroup definition; [2]components of the JGL definition; [3]components of the PGAM definition.

ACQ-5, Asthma Control Questionnaire 5-item; CFB, change from baseline; CR, clinical remission; FEV_1, forced expiratory volume in 1 s; FF, fluticasone furoate; ITT, intention-to-treat; JGL, Japanese Guidelines for adult asthma; PGAM, Practical Guidelines for Asthma Management;
UMEC, umeclidinium; VI, vilanterol.

Supplementary Table S6. Proportion of participants meeting the CR criteria (Week 24) or achieving CR (Week 52) by individual component (non-cumulative) (long-term safety study)

**Patients,** n (%)	**FF100/UMEC/VI (N=47)**	**Switched FF/UMEC/VI (N=9)**	**FF200/UMEC/VI (N=55)**
**Week 24**			
**SCS free**[1,2,3]	43 (91)	7 (78)	47 (85)
**Severe exacerbation free**[1,2,3]	43 (91)	7 (78)	47 (85)
**ACQ-5 total score <1.5**[1]	43 (91)	6 (67)	35 (64)
**ACQ-5 total score ≤0.75**[2,3]	20 (43)	0	16 (29)
**CFB in trough FEV_1 ≥100 mL**[1,2]	31 (66)	4 (44)	22 (40)
**CFB in trough FEV_1 ≥0 mL**[1,2]	40 (85)	4 (44)	40 (73)
**Week 52**			
**SCS free**[1,2,3]	42 (89)	5 (56)	42 (76)
**Severe exacerbation free**[1,2,3]	42 (89)	5 (56)	44 (80)
**ACQ-5 total score <1.5**[1]	39 (83)	6 (67)	37 (67)
**ACQ-5 total score ≤0.75**[2,3]	20 (43)	3 (33)	19 (35)
**CFB in trough FEV_1 ≥100 mL**[1,2]	28 (60)	3 (33)	23 (42)
**CFB in trough FEV_1 ≥0 mL**[1,2]	36 (77)	5 (56)	38 (69)

All doses are in mcg. [1]Components of the Workgroup definition; [2]components of the JGL definition; [3]components of the PGAM definition.

ACQ-5, Asthma Control Questionnaire 5-item; CFB, change from baseline; CR, clinical remission; FEV_1, forced expiratory volume in 1 s; FF, fluticasone furoate; JGL, Japanese Guidelines for adult asthma; PGAM, Practical Guidelines for Asthma Management; SCS, systemic corticosteroid; UMEC, umeclidinium; VI, vilanterol.
